# Detection and Correlation of Single and Concomitant *TP53*, *PTEN,* and *CDKN2A* Alterations in Gliomas

**DOI:** 10.3390/ijms20112658

**Published:** 2019-05-30

**Authors:** Igor Andrade Pessôa, Carolina Koury Amorim, Wallax Augusto Silva Ferreira, Fernanda Sagica, José Reginaldo Brito, Moneeb Othman, Britta Meyer, Thomas Liehr, Edivaldo Herculano C. de Oliveira

**Affiliations:** 1 Programa de Pós-graduação em Genética e Biologia Molecular, Universidade Federal do Pará, Belém, PA 66075-110, Brazil; igora.pessoa@hotmail.com; 2 Programa de Pós-graduação em Neurociências e Biologia Celular, Universidade Federal do Pará, Belém, PA 66075-110, Brazil; wallaxaugusto@gmail.com; 3 Laboratório de Cultura de Tecidos e Citogenética, SAMAM, Instituto Evandro Chagas, Ananindeua, PA 67030-000, Brazil; carolinaamorim@iec.gov.br (C.K.A.); fernandasagica@iec.gov.br (F.S.); 4 Programa de Pós-graduação em Oncologia e Ciências Médicas, Universidade Federal do Pará, Belém, PA 66075-110, Brazil; jrnbrito@gmail.com; 5 Institute of Human Genetics, Jena University Hospital, Am Klinikum 1, 07747 Jena, Germany; Moneeb.Othman@med.uni-jena.de (M.O.); Thomas.Liehr@med.uni-jena.de (T.L.); 6 Zyto Vision GmbH, 27572 Bremerhaven, Germany; meyer@zytovision.com; 7 Faculdade de Ciências Naturais, ICEN, Universidade Federal do Pará, Belém, PA 66075-110, Brazil

**Keywords:** glioma, *TP53*, *PTEN*, *CDKN2A*, co-alterations, single-strand conformational polymorphism, array-comparative genomic, hybridization and fluorescence in situ hybridization

## Abstract

Gliomas are the most frequent primary tumors of central nervous system and represent a heterogeneous group of tumors that originates from the glial cells. *TP53*, *PTEN*, and *CDKN2A* are important tumor suppressor genes that encode proteins involved in sustaining cellular homeostasis by different signaling pathways. Though genetic alterations in these genes play a significant role in tumorigenesis, few studies are available regarding the incidence and relation of concomitant *TP53*, *PTEN*, and *CDKN2A* alterations in gliomas. The purpose of this study was to evaluate the occurrence of mutation and deletion in these genes, through single-strand conformational polymorphism, array-comparative genomic hybridization, and fluorescence in situ hybridization techniques, in 69 gliomas samples. Molecular results demonstrated a significant higher prevalence of *TP53, PTEN,* and *CDKN2A* alterations in astrocytoma than other tumor subtypes, and heterozygous deletion was the most frequent event. In addition, a significant association was observed between *TP53* and *CDKN2A* alterations (*p* = 0.0424), which tend to coexist in low grade astrocytomas (5/46 cases (10.9%)), suggesting that they are early events in development of these tumors, and *PTEN* and *CDKN2A* deletions (*p* = 0.0022), which occurred concomitantly in 9/50 (18%) patients, with *CDKN2A* changes preceding *PTEN* deletions, present preferably in high-grade gliomas.

## 1. Introduction

Glioma is a general term used to characterize tumors originated from glial brain cells [1]. They are the most frequent primary tumors of the central nervous system (CNS), and are characterized for a heterogeneous set of diseases, ranging from benign to malignant tumors, with a diverse array of histology and molecular differences and clinical outcomes [2,3]. Historically, gliomas have been diagnosed and classified based on histopathology, according to their microscopic characteristics and clinical behavior. Until recently, these tumors were classified, according to the World Health Organization (WHO) classification system, into astrocytomas, oligodendrogliomas, oligoastrocytomas, and ependymomas depending on their cells of origin [4]. However, new molecular markers have emerged in recent decades, and these findings have contributed to a better understanding of glioma biology, providing new information concerning tumor initiation, progression, and clinical outcome [5]. Hence, in 2016, the WHO classification was updated, and, for the first time, it integrated classic histological features with molecular biomarkers to define distinct gliomas entities, interrupting the idea of only using histopathological analyses as the criterion for definition and classification of CNS tumors [6]. 

Among molecular markers, there are several tumor suppressors genes, such *TP53*, *PTEN,* and *CDKN2A*, which are involved in sustaining cellular homeostasis by different signaling pathways, having been reported to be altered in different human cancers [7]. *TP53* is a tumor suppressor gene localized on chromosome site 17p13.1, and encodes the p53 protein, a transcription factor involved in regulation of multiple cell functions implicated in cancer biology, such as regulation of cell proliferation, DNA repair, apoptosis, and differentiation [8]. *PTEN*, located on chromosome site 10q23.3, is a tumor suppressor gene that encodes a 403-amino acid polypeptide, which is a dual lipid/protein phosphatase considered essential for tumor suppressor properties, since its phosphatase activity makes it a negative regulator of PI3K and Akt/mTOR signaling, and thus playing important roles in the regulation of apoptosis, cell cycle arrest, angiogenesis, adhesion and invasion, and DNA damage repair [9,10]. *CDKN2A* gene is localized on chromosome site 9p21, being a negative G1 cell-cycle regulatory gene [11,12], since it encodes both p16INK4a and p14ARF proteins [13,14]; p16 is a tumor suppressor protein that induces cell-cycle arrest by inhibition of cyclinD-CDK4 and cyclinD-CDK6 complexes, indicating the phosphorylation of Rb protein [15], while p14ARF is a protein that blocks Mdm2-mediated degradation of p53 [16]. 

It has been showed that mutations and/or deletions of tumor suppressor genes are critical events behind the pathogenesis of gliomas [17]. Hence, considering the importance of *TP53, PTEN,* and *CDKN2A* genetic alterations in different proposals of initiation, progression, and classification of gliomas, the aim of this study was to analyze the occurrence of allelic deletions in these genes, as well as to perform a screening of *TP53* and *PTEN* gene mutations in 69 samples of gliomas. Through this study, we have described the prevalence pattern of individual and mutually genetic alterations of these three genes in these gliomas and established their possible associations with clinical variables such gender, age, histological types, and WHO histological grading

## 2. Results

### 2.1. Clinical Parameters

Among the 69 gliomas samples analyzed, 41 (59.4%) were males and 28 (40.6%) females, with mean age at diagnosis of 35.1 years (ranging from 1 to 79 years). We compared the mean age distribution among different WHO grades of malignancy by analysis of variance (ANOVA) and identified statistically significant differences in younger patients with less aggressive tumors, whereas the increase of mean age was accompanied by an increase in tumor aggressiveness (Table 1).

### 2.2. Molecular Data

#### 2.2.1. *TP53* Status

**Figure 1:**
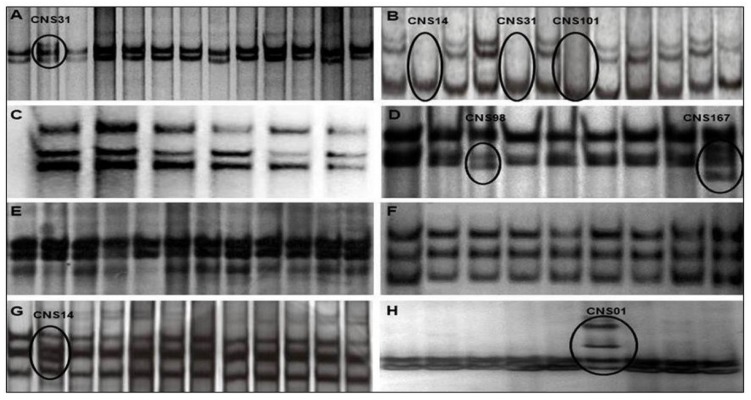
*TP53* gene SSCP. The results show in the images (**A**), (**B**), (**D**), (**G**), and (**H**) show that some samples presented aberrantly migrated bands in the SSCP, representing the exons 4, 5, 7, 10, and 11 respectively, indicating the presence of changes in these regions, while the analysis of samples for the other exons revealed a monomorphic pattern of migration, as we can see in the images (**C**), (**E**), and (**F**), which represent the exons 6, 8, and 9, respectively.

*TP53* mutational status (exons 4–11) was determined in 48 cases, while 65 were successfully analyzed for *TP53* deletion evaluation and 44 for both analyses. Of 48 gliomas samples analyzed, PCR-SSCP revealed aberrantly migrated bands in 6 (12.5%) (Table 2). Among these six samples, a total of eight mutations were identified, with exon 5 being the most altered, mutated in three cases (50%), followed by exon 7, mutated in two cases (33.3%) and exons 4, 10, and 11, mutated in one case each (16.7%). The results for each exon are shown in Figure 1. 

*TP53* mutation was more prevalent in male patients (66.7%) and the mean age of patients with mutation was 44.2 years, higher than wild-type *TP53* patients (35.5). However, no significant association was found between the presence of *TP53* mutation and gender and age (*p* = 0.6441 and *p* = 0.2143, respectively). A statistically significant prevalence of this alteration in astrocytomas was observed, since only this subgroup presented mutations (*p* = 0.0016). In addition, WHO grades II and IV showed highest frequency of *TP53* mutations in the study, since 33.3% (2/6) of the changes were identified in each one of them. However, no association was observed between the presence of mutation and grade of malignancy (*p* = 0.9985).

**Figure 2:**
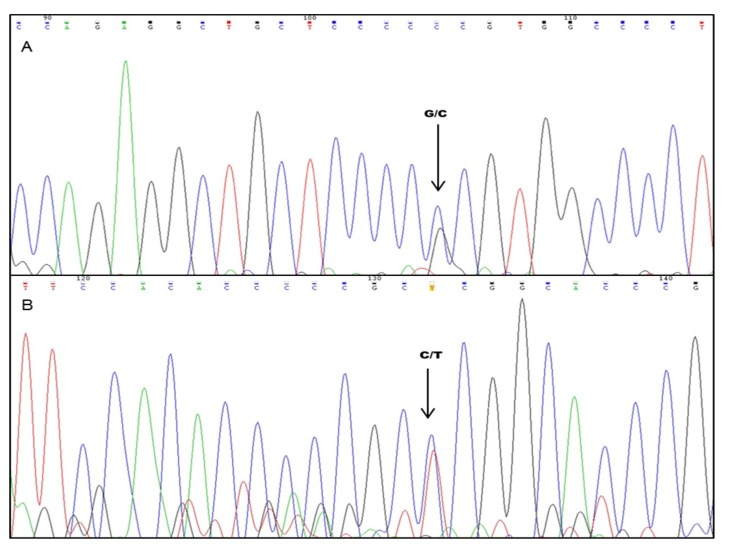
Nucleotide changes identified in *TP53*. (**A**) G→C polymorphism at codon 72 of exon 4 (Arg/Pro) (**B**) C→T transition mutation at codon 153 of Exon 5 (Leu/Pro).

Sequencing was used for validation of SSCP results in one case (CSN 31—anaplastic astrocytoma). In exon 4, the codon 72 polymorphism (rs = 1,042,522), which results in a G→C transversion in the second position of the codon, resulted in the substitution of Arginine for Proline. In exon 5, a C→T mutation was identified at codon 153 (position 458 of the coding region—COSM44367), which change the amino acid Proline to Leucine. The sample presented the genotype in heterozygous, Arg/Pro (G/C) and Pro/Leu (C/T), respectively (Figure 2).

**Figure 3:**
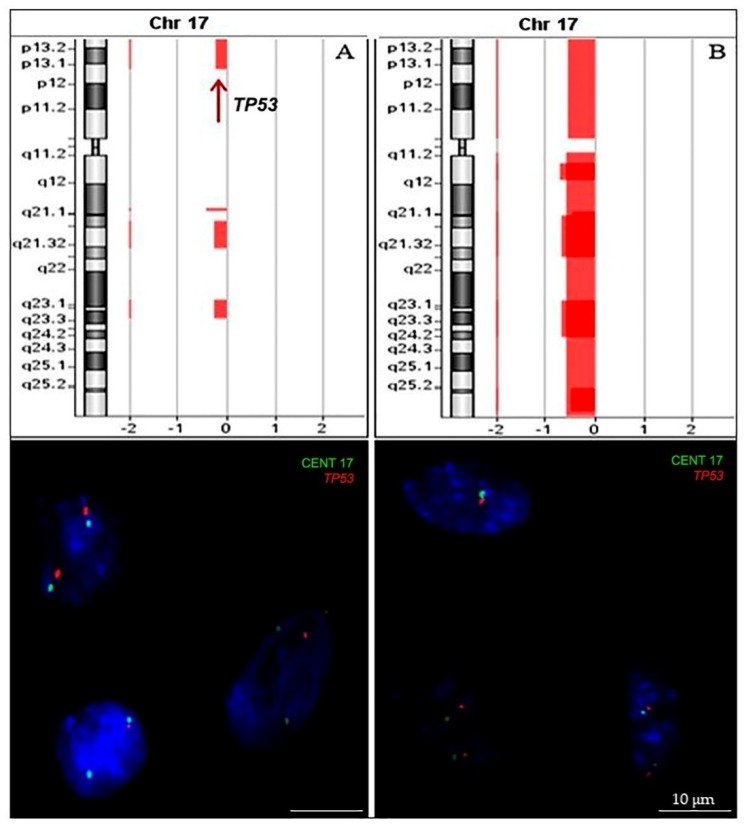
*TP53* allelic loss identified by array-comparative genomic hybridization (CGH) (upper line) was confirmed by interphase fluorescence in situ hybridization (iFISH) using LSPs (lower line). Results for two exemplary cases are shown: Image (**A**) shows heterozygous deletion of *TP53* gene; image (**B**) shows monosomy of chromosome 17. Scale bars = 10 µm.

Analysis of *TP53* deletion was performed in 65 of the cases, 18 of which were analyzed by array-comparative genomic hybridization (aCGH) alone, 23 by interphase fluorescence in situ hybridization (iFISH), and 24 by both methods. Figure 3 illustrate *TP53* deletion identified by array-CGH and confirmed by iFISH. There was a general agreement between aCGH and FISH methodologies, with concordance for detection of *TP53* deletion of 91.7%. In two cases, *TP53* deletion was identified by iFISH, but not by aCGH. Of the 65 cases analyzed, heterozygous deletion was identified in 19 cases (29.2%). No homozygous deletions were found in this study. 

*TP53* deletion was slightly more prevalent in female patients (52.6%) and the mean age of patients harboring this alteration (39.7 years) was higher than those with no deletion (35.5 years). However, no significant association was found between the presence of this abnormality and age (*p* = 0.1835). The frequency of *TP53* deletion was significantly higher in astrocytomas (15/49 cases, 30.6%) when compared to other histological groups (*p* = 0.0048). Further, the highest frequency of *TP53* deletion was identified in high-grade gliomas (HGG) (WHO grade III and IV) when compared to low-grade gliomas (LGG) (WHO grade I and II) (13/37 cases, 35.1% vs. 6/28 cases, 21.4% respectively); however, no association was observed between the presence of this alteration and WHO grading groups (*p* = 0.3535). Table 3 summarizes the frequency of *TP53* deletion for each histologic subtype and WHO grading groups.

The occurrence of concomitant allelic deletion and mutation in *TP53* gene were analyzed in a total of 44 samples, and it was observed that in the 4 samples that harbored *TP53* mutations, 2 of them (50%) also presented *TP53* heterozygous deletion. However, no significant association between presence of *TP53* mutation and allelic loss was found (*p* = 0.2530). 

#### 2.2.2. *PTEN* Status

**Figure 4:**
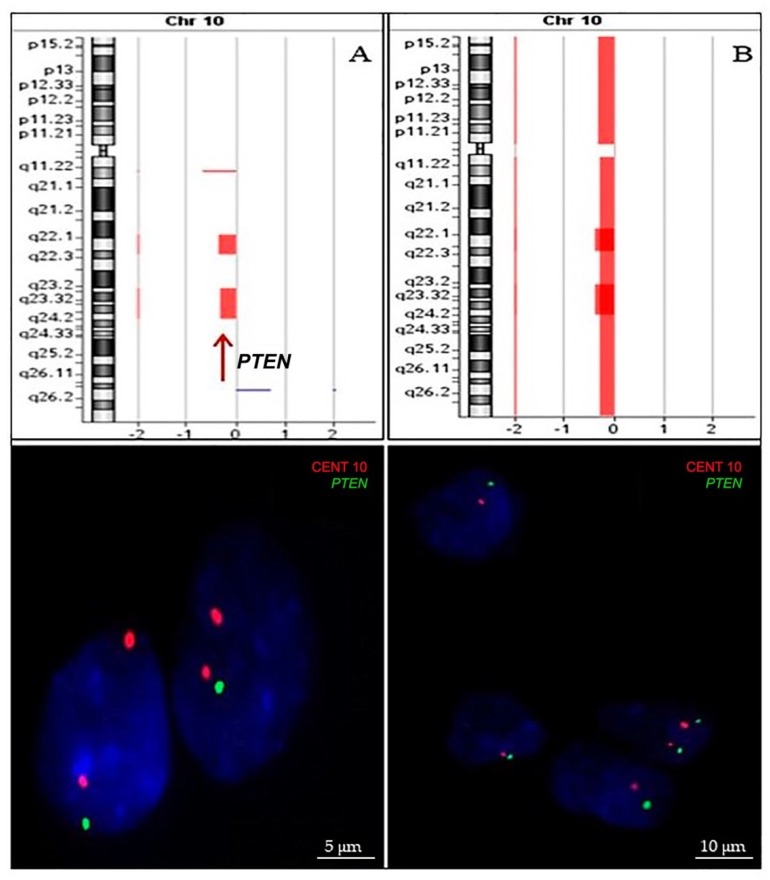
*PTEN* allelic loss identified by array-CGH (upper line) was confirmed by iFISH using LSPs (lower line). Results for two exemplary cases are shown: Image (**A**) shows heterozygous deletion of *PTEN* gene; image (**B**) shows monosomy of chromosome 10.

*PTEN* mutational status (exons 5–7) was determined in 34 cases, while 62 were successfully analyzed for *PTEN* deletion evaluation and 32 for both analyses. No aberrantly migrating bands were observed by PCR-SSCP. Among the 62 samples that were evaluated for *PTEN* deletion, 37 were analyzed by aCGH alone, 20 by iFISH, and 5 by both methods. Figure 4 illustrate *PTEN* deletion identified by array-CGH and confirmed by iFISH. There was agreement between aCGH and FISH methodologies, with concordance for detection of *PTEN* deletion of 100%. Of the 62 cases analyzed, heterozygous deletion was identified in 23 cases (37.1%). No homozygous deletions were found in this study. 

**Figure 5:**
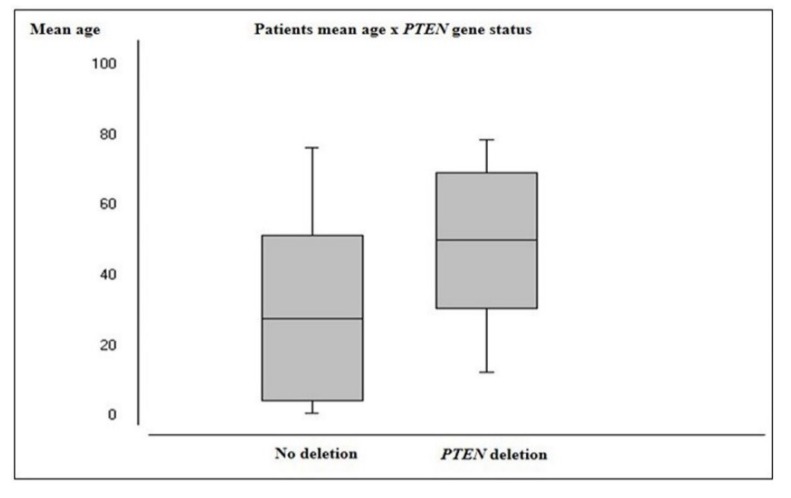
Patients’ mean age according to *PTEN* gene status.

*PTEN* deletion was more prevalent in male patients (69.6%) and the mean age of patients harboring this alteration (50.5 years) was significantly higher than those with no deletion (28.3 years; *p* = 0.0002) (Figure 5). Astrocytomas demonstrated a significantly higher frequency of *PTEN* deletion (20/46 cases, 43.5%) than other histological groups (*p* ≤ 0.0001). Additionally, HGG presented significantly higher frequencies of *PTEN* deletion (20/36 cases, 55.6%) than LGG (3/26 cases, 11.5%) (*p* = 0.05). Table 4 summarizes the frequency of *PTEN* deletion for each histologic subtype and WHO grading groups.

#### 2.2.3. *CDKN2A* Status

**Figure 6:**
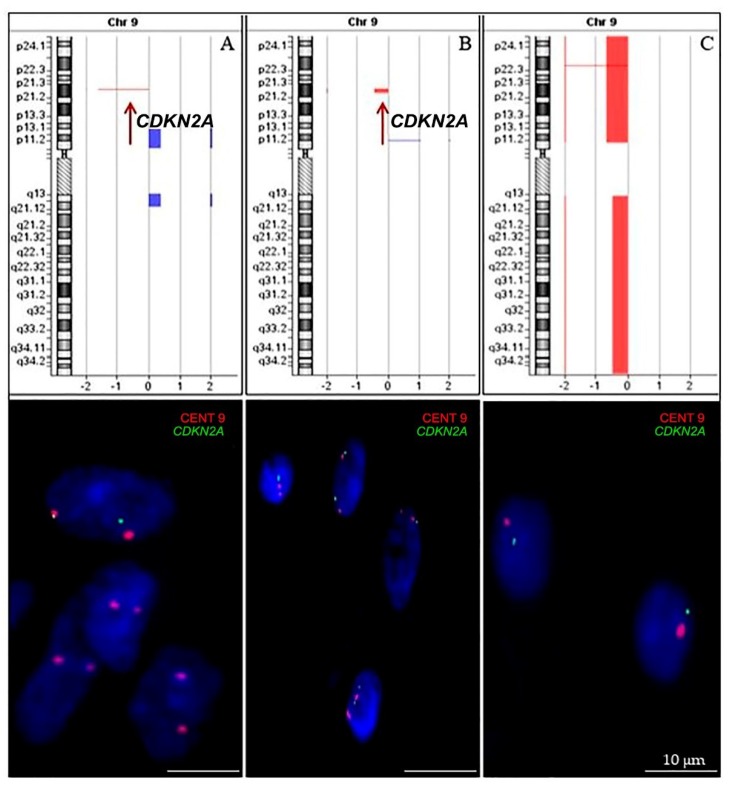
*CDKN2A* allelic loss identified by array-CGH (upper line) was confirmed by iFISH using LSPs (lower line). Results for three exemplary cases are shown: Image (**A**) shows homozygous deletion of *CDKN2A* gene; image (**B**) shows heterozygous deletion of *CDKN2A* gene; image (**C**) shows monosomy of chromosome 9. Scale bars = 10 µm.

A total of 63 samples were evaluated for *CDKN2A* deletion, of which 36 were analyzed only by aCGH, 21 by iFISH, and 6 by both methods. Figure 6 illustrate *CDKN2A* deletion identified by array-CGH and confirmed by iFISH. There was agreement between aCGH and FISH methodologies, with concordance for detection of *CDKN2A* loss of 100%. Of the 63 cases analyzed, *CDKN2A* deletion was identified in 28 cases (44.4%). A total of 20 samples (71.4%) showed heterozygous deletion, while homozygous deletions were found in 8 samples (28.6%).

**Figure 7:**
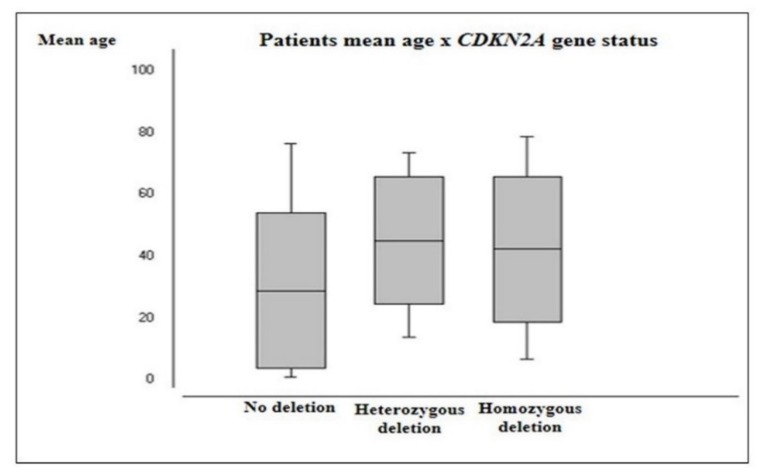
Patients’ mean age according to *CDKN2A* gene status.

*CDKN2A* deletion was more prevalent in male patients (71.4%) and the mean age of patients harboring heterozygous deletion (45.5 years) and homozygous deletions (42.6 years) were significantly higher than those with no deletion (29.2 years; *p* = 0.0091 and *p* = 0.0089, respectively) (Figure 7). Astrocytomas demonstrated a significantly higher frequency of both *CDKN2A* heterozygous (16/47 cases, 34%) and homozygous deletion (8/47 cases, 17%) than other histological groups (*p* ≤ 0.0001 and *p* = 0.006, respectively). Moreover, HGG presented a higher frequency of heterozygous (14/37 cases, 37.8%) and homozygous deletion (6/37 cases, 16.2%) than LGG (6/26 cases, 23.1% and 2/26 cases, 7.7%, respectively). However, no significant association was observed between the presence of this alteration and WHO grading groups (*p* = 0.1901). Table 5 summarizes the frequency of *CDKN2A* deletion for each histologic subtype and WHO grading groups.

### 2.3. Frequency and Association between TP53, PTEN, and CDKN2A Genetic Alterations

**Figure 8:**
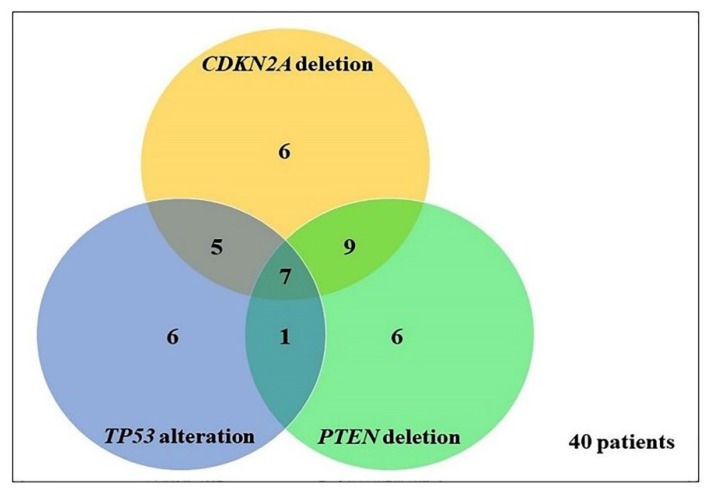
Association of *TP53*, *PTEN*, and *CDKN2A* alteration in 40 gliomas.

Concurrent *TP53*, *PTEN,* and *CDKN2A* analyses were available in 62 samples, of which 40 (64.5%) presented alteration in at least one gene (Figure 8). *TP53* alteration (mutation or deletion), *PTEN,* and *CDKN2A* deletion occurred together in seven samples, 6/46 (13%) astrocytomas, and 1/4 (25%) oligodendrogliomas. The simultaneous occurrence of alterations in two genes was observed as follows: *PTEN* and *CDKN2A* genes, identified in nine patients, 8/46 (17.4%) astrocytomas, and 1/4 (25%) oligoastrocytoma; *TP53* and *CDKN2A* genes, identified in 5/46 (10.9%) astrocytomas; and *TP53* and *PTEN* genes, identified in 1/46 (2.2%) astrocytomas. Considering cases with alterations in only two genes, a significant association was observed between *TP53* and *CDKN2A* alterations (*p* = 0.0424) and between *PTEN* and *CDKN2A* deletions (*p* = 0.0022), but not between *TP53* and *PTEN* alterations (*p* = 0.5879).

Table 6 summarizes the frequencies of alterations per WHO CNS grading. A significant association was detected between the presence of concurrent alterations and WHO grade (*p* = 0.0408), since the increase in grade of malignancy was accompanied by an increase in the number of concomitant gene alterations. Co-alterations of *TP53* and *PTEN* genes were present in only HGG (2.8%), and combined *PTEN* and *CDKN2A* alterations were observed predominantly in HGG (22.2%) when compared to LGG (3.8%). Additionally, co-alteration in *TP53* and *CDKN2A* genes were also more frequently observed in HGG, however, with a frequency similar to the one observed in LGG (8.3% vs. 7.7%). Further, a higher frequency of concurrent *TP53*, *PTEN,* and *CDKN2A* alterations was observed in gliomas of higher malignancy grade (16.7%). 

## 3. Discussion

Over the last decades, our understanding of the genetic profile of CNS tumors has improved considerably. Large-scale cytogenetic and molecular studies have detected many recurrent genetic and epigenetic abnormalities associated with different subtypes of glial tumors, and it has become clear that these markers are useful in identifying more uniform gliomas subgroups [18]. Like in other types of cancers, initiation and progression of gliomas are a consequence of sequential genetic alterations, such as point mutations, epigenetic changes, copy number alterations, and chromosomal rearrangements, affecting genes that are essential to maintaining cellular homeostasis [19]. Therefore, the focus of this investigation was to analyze the influence of independently and combined alterations in *TP53*, *PTEN,* and *CDKN2A* tumor suppressor genes in the gliomagenesis, evaluating a possible association of our findings with clinical variable of the patients.

Genetic alterations in *TP53*, *PTEN,* and *CDKN2A* genes are important molecular markers, being commonly observed in gliomas [20]. Loss of function of their respective proteins, which are inactivated directly by mutations and allelic deletions or indirectly by alterations of genes that interact with their protein product, is a common characteristic in a majority of human cancers, resulting in escape from the tumor-suppressor process [20]. 

In our study, *TP53* abnormalities (mutation and/or deletion) occurred in 23/69 (33.3%) samples, with a significant prevalence of both gene mutation (*p* = 0.0016) and allelic loss (*p* = 0.0048) in astrocytomas, when compared to the other histological groups, confirming previous reports [21,22]. *TP53* deletions were more frequent than mutations and were detected in 19/65 cases (29.2%) and 6/48 cases (12.5%), respectively, confirming the data of previous publications demonstrating higher incidence of *TP53* deletion than gene mutation in cancer [23,24,25]. In addition, no significant association (*p* = 0.2530) between presence of mutation and deletion of *TP53* was found in the 44 samples in which both analyses were allowed.

Since most *TP53* mutations (~90%) identified in gliomas are concentrated in mutational hotspots regions (exons 5–9), studies have limited their mutational analysis to this portion of the gene. This fact has caused some bias in the literature, because mutations located outside these classic hotspots have already been identified, especially in primary glioblastoma [26]. In an attempt to avoid this bias, we performed a screening of mutations in a region that included exons 4 to 11 of the gene. In our study, we found that 5 of 8 (62.5%) mutations in *TP53* gene were localized into hotspot region, confirming the prevalence of mutations described in literature in this region; however, 3 mutations (37.5%) were present in exons 4, 10, and 11, thus showing the need for studying all exons of *TP53* gene in gliomas.

On the other hand, although some reports have identified low frequency of *PTEN* mutations in HGG [27,28], which is consistent with our results, since no mutation was identified in 34 tumors, a mutational rate in up to ~60% in high-grade astrocytomas has been detected in most of the previous studies [29,30,31]. A sampling bias can explain this divergence with our findings, since the heterogeneity of tumor subtypes and sample size in our study may have influenced these conflicting results. Further, other genetic events may be associated with inactivation of the PTEN protein and consequent participation in the process of malignant progression of gliomas. *PTEN* deletions also have been reported in up to 88.1% of anaplastic astrocytomas, from 0% to 50% in oligodendrogliomas/oligoastrocytomas and rarely in LGG [32,33,34,35,36,37]. Consistent with these studies, we found the presence of *PTEN* deletion in 23 cases from 62 (37.1%), with a significantly higher prevalence detected in astrocytomas (*p* ≤ 0.0001). Although most investigations show a higher frequency of *PTEN* mutation when compared to allelic loss [18,38,39], Pollack et al. performed a sequence analysis of the nine exons of *PTEN* in a series of 62 malignant gliomas and found mutation in only one case, while *PTEN* allelic imbalance was detected in 7 of 22 cases (31.8%) [40], which is consistent with our findings.

Besides the alterations described above, high occurrence of losses involving *CDKN2A* gene have been reported in gliomas, ranging from 11% to 71% in all histological types, although more frequently found in astrocytomas [41,42,43]. Our findings are consistent with literature, since we report *CDKN2A* deletions in 28 from 63 cases (44.4%), with significantly higher frequency of both heterozygous and homozygous deletion in astrocytomas (*p* ≤ 0.0001 and *p* = 0.006 respectively). 

Previous publications have shown that *TP53* heterozygous deletion occur more frequently than homozygous deletion in cancer [44,45]. Our findings corroborate these observations, since we detected only heterozygous deletion in *TP53* gene. Contradicting previous studies reporting higher frequency of homozygous deletions of *PTEN* and *CDKN2A* genes in gliomas [18,34,41,42], in our study only *PTEN* heterozygous deletions were found, while genomic analyses of *CDKN2A* showed that 28 patients harboring this alterations: 71.4% presented heterozygous deletion against 28.6% showing homozygous deletions. 

Although *TP53*, *PTEN,* and *CDKN2A* are considered recessive tumor-suppressor genes, and deletions are frequently related with mutations of the second allele, supporting the “two-hit” of cancer development [46], many studies have shown the effect of their inactivation by a heterozygous mutation or one allele loss on tumorigenesis [25]. It has been proposed that cell proliferation rates can be increased by haploinsufficiency of tumor-suppressor genes and that heterozygous deletions in these genes are supposed to be preferentially selected during tumor progression [47,48]. 

Studies using mouse models have shown that *PTEN* heterozygous deletion promotes genomic instability and leads to preferential rearrangements at fragile sites [49]. In addition, it was reported that loss of both *TP53* alleles is not a prerequisite for tumor formation and progression, since analyses of tumors in heterozygous animals showed that many of the tumors preserved a wild-type *TP53* allele [25]. Moreover, analyses of human cancers samples obtained from patients diagnosed with the Li-Fraumeni syndrome have shown that the remaining wild-type *TP53* allele is not always lost, proposing that haploinsufficiency of *TP53* may be enough for tumor initiation [50]. In addition, Picanço-Albuquerque et al. showed in a series of 18 biopsies of prostate cancer that two cases with a heterozygous *PTEN* deletion detected by FISH presented decreased expression of PTEN protein [47]. Thus, it is thought that a heterozygous deletion with retention of a *PTEN* allele intact may reduce the protein expression levels. 

Overall, it has been reported that *TP53* genetic alterations are early events in gliomagenesis, while genetic changes affecting *PTEN* have been identified mainly in HGG, considered to be involved in malignant glioma progression. Our data are in concordance previous studies, since we found no statistically significant difference between the presence of *TP53* mutation/deletion and grade of malignancy (*p* = 0.9985 and *p* = 0.3535, respectively), while a significantly higher frequency of *PTEN* deletion in HGG was identified (55.6%) (*p* = 0.05). In addition, although the majority of investigations have shown that *CDKN2A* deletions are more frequently identified in HGG when compared to LGG, suggesting a role for *CDKN2A* pathway in malignant progression [41,42], no significant association was observed between the presence of *CDKN2A* losses and WHO grading groups (*p* = 0.1901) in our study, suggesting that this is an early event in initiation and progression of gliomas. Our results are supported by Reis et al., who reported no significant differences when the frequency of *CKN2A* deletion was compared between low- and high-grade tumors (44.7% × 50.8%; p = 0.6, respectively) [42], and also by Purkait et al., who found lower frequency of *CDKN2A* deletion in grade-II tumors (27.3%) when compared to grade-III tumors (30%), although the difference was not statistically significant [41].

In addition, in order to validate our findings, we investigated the frequency of these specific alterations in large-scale available glioma database (http://www.cbioportal.org), which includes TCGA (The Cancer Genome Atlas) datasets. Thus, we compiled the data obtained and compared the frequencies with our results. In general, the alterations pattern described in our analysis correspond to that found in these datasets. While *PTEN* alterations (mutation and deletion) were detected predominantly in HGG (346/1310 cases, 26.4%) when compared to LGG (29/847 cases, 3.4%), the frequency of *TP53* alterations was similar in these two groups (LGG (350/847 cases, 41.3% against HGG (426/1310 cases, 32.5%)), supporting our experimental data. On the other hand, concerning *CDKN2A* deletion, we found a different situation, since this alteration was found mainly in HGG (426/2114 cases, 53.9%), while in LGG was identified in 8.9% (67/750) of samples, suggesting that this is a late event in progression of gliomas and is involved in tumor malignancy. However, as previously mentioned, published data on *CDKN2A* deletion in gliomas are still conflicting, and further studies are needed to clarify the timing of changes in this gene in the progression of glial tumors. It is also important to report that, according to the TCGA dataset, *TP53*, *PTEN,* and *CDKN2A* alterations (mutation and deletion) are more prevalent in gliomas derived from astrocytic lineage, which confirms our results, since we found a significant association of these genes alterations with astrocytomas. 

Most of our glioma samples harbored alterations in at least one gene (40/62 cases, 64.5%). The most frequent aberrations detected were concomitant deletion of *PTEN* and *CKDN2A* genes (9/40 cases, 22.5%), followed by simultaneous alterations in all three genes (7/40 cases, 17.5%), individual changes in *TP53*, *PTEN,* and *CDKN2A* (6/40 cases each, 15%), co-alteration of *TP53* and *CKDN2A* (5/40 cases, 12.5%), and co-alteration of *TP53* and *PTEN* genes (1/40 cases, 2.5%). Stankovic et al. had similar findings, since in their analyses of 30 glioma samples, 27 presented abnormalities in either one of *TP53*, *PTEN,* and *CDKN2A* genes (90%) [7]. In addition, among these 27 samples that showed alterations, it was observed a higher frequency of co-alteration in *PTEN* and *CDKN2A* genes (29.6%), followed by alterations of all three genes (18.5%), co-alteration in *TP53* and *CDKN2A* (14.8%), and concomitant deletion of *TP53* and *PTEN* genes (3.7%). 

Overall, a strong association was noted for the co-occurrence of *PTEN* and *CDKN2A* deletions (*p* = 0.0022). Similarly, significant association was found for the co-alterations of *TP53* and *CDKN2A* (*p* = 0.0424). However, no significant association was detected between *TP53* and *PTEN* alterations (*p* = 0.5879). These findings are similar to other studies that found an association for the co-occurrence of *PTEN* and *CDKN2A* deletions [7,33] and/or *TP53* and *CDKN2A* alterations in gliomas [15,51], while concomitants *TP53* and *PTEN* alterations were less frequent [7,52,53]. 

When tumor grade was evaluated, a significant increase in the number of co-occurrence of genetic alterations in the three genes was detected, according to increase of WHO grade (*p* = 0.0408), and supporting previous reports [33,37], suggesting that combined alterations are involved in malignant tumor progression. Although it was observed that co-occurrence of *PTEN* and *CDKN2A* losses (both heterozygous and homozygous deletion) were detected in higher frequency in HGG, LGG showed a frequency of individual *CDKN2A* deletion similar to that observed in more aggressive tumors, while the presence of individual *PTEN* deletion was significantly higher in more aggressive tumors, suggesting that allelic loss of *CDKN2A* is a prior event to *PTEN* alterations. 

Although much have been reported on the presence of genetic alterations in *TP53*, *PTEN,* and *CDKN2A* genes in several tumor types, few studies have focused on the analysis of the incidence of co-alterations of these three genes, through different types of mechanisms which lead to loss of protein function, such as mutations and deletions, in a single sample of gliomas. Our results demonstrated the presence of simultaneous alterations of these genes and association with different clinical-pathological variables of the patients, evidencing its importance in the development and progression of gliomas. In summary, all the alterations detected in our study were associated with astrocytic phenotype and older age. In addition, an important finding of our results is the higher prevalence of heterozygous deletion when compared to homozygous deletion, and since these changes have rarely occurred simultaneously with mutations in these genes, which could inactivate the second allele, supporting the “two-hit” idea of cancer development, we suggest that heterozygous allele loss are fundamental in the development of the tumors analyzed. Further, our findings indicate that *TP53* changes and *CDKN2A* deletions tend to coexist in LGG, suggesting that they are early events in progression of these tumors, as well as *CDKN2A* and *PTEN* deletions, since a significant association was observed, with *CDKN2A* changes preceding *PTEN* deletions, that are involved with malignant progression of gliomas. 

## 4. Materials and Methods 

### 4.1. Patients and Tissue Samples

All subjects gave their informed consent for inclusion before they participated in the study. The study was conducted in accordance with the Declaration of Helsinki, and the protocol was approved by the Ethics Committee of Instituto Evandro Chagas (Belém, Brazil) under the code CAA 01698912.7.3002.5550 (11 April 2014). Tumor samples were obtained fresh at the time of surgery at the Hospital Ophir Loyola (Belém, PA, Brazil) and transported to the Laboratório de Cultura de Tecidos e Citogenética in Instituto Evandro Chagas (IEC) (Ananideua, PA, Brazil). Biopsies were divided in two parts and used for DNA extraction and cell culture. 

The samples were collected before the publication of the updated WHO classification in 2016. Clinical features of patients’ samples are shown in Table 7. All cases were histologically graded and classified according to the 2007 WHO classification for CNS tumors. We examined a total of 69 gliomas, including 2 subependymal giant cell astrocytoma (WHO grade I), 9 pilocytic astrocytomas (WHO grade I), 2 pilomyxoid astrocytoma (WHO grade II), 1 pleomorphic xanthoastrocytoma (WHO grade II), 9 diffuse astrocytomas (WHO grade II), 6 anaplastic astrocytomas (WHO grade III), 22 gliobastomas (WHO grade IV), 2 gliosarcomas (WHO grade IV), 2 oligoastrocytoma (WHO grade II), 2 anaplastic oligoastrocytomas (WHO grade III), 3 oligodendrogliomas (WHO grade II), 1 anaplastic oligodendrogliomas (WHO grade III), 4 ependymomas (WHO grade II), and 4 anaplastic ependymomas (WHO grade III). Clinical parameters including age and gender were collected. 

### 4.2. DNA Extraction

High molecular weight DNA was isolated from the tumor samples using the Illustra Tissue and Cells GenomicPrep Mini Spin Kit (GE Healthcare Life Science, (Little Chalfont, Buckinghamshire, UK). The concentration and purity of DNA was evaluated by agarose gel electrophoresis and by Nanodrop ND-2000 Spectrophotometer (Thermo Scientific, Waltham, Massachusetts, USA). The absorbance ratio at 260 nm and 280 nm, as well as the ratio absorbance at 260 nm and 230 nm, were analyzed as measures of quantity and quality of the DNA extracted. 

### 4.3. Single-Strand Conformational Polymorphism (SSCP) Analysis

Exons 4–11 of *TP53* gene and 5–7 of *PTEN* gene were separately amplified by PCR using specific sets of primers (Table 8 and Table 9). The primers used in this study were developed with support of the specialized software programs Primer3, Blat, and AutoDimer. PCR reactions were carried out in a total volume of 25 μL, using concentrations of 0.1 μM of deoxynucleotide triphosphate, 1.5 μM of MgCl2, 0.1 μM of each of the primers (forward and reverse), 50 mM of KCl, 1 U of Taq polymerase, and 100 ng/μL of sample DNA. PCR conditions for the genes amplification were: Initial denaturation at 95 °C for 5 min, 35 cycles at 95 °C for 1 min, annealing at specific temperature for each primer (Table 1) for 1 min, and 72 °C for 50 s, with a final extension of 8 min at 72 °C. PCR reactions were analyzed in 1.5% nondenaturing agarose gel. 

After PCR amplification, the products were submitted to a 95 °C denaturing condition for 5 min and stabilized the single strands with a thermal shock on ice. Electrophoresis in a polyacrylamide gel with different conditions of concentration, voltage, and time of running were performed for each exon, and then silver-stained and prepared to be analyzed. In order to validate the efficiency of SSCP, we decided to sequence one of the positive cases for mobility shifts in polyacrylamide gel. The PCR product was sequenced using the Big Dye Terminator kit (v3.1, Applied Biosystems, Foster City, CA, USA) following manufacturer specifications. The ABI PRISM 3130 Genetic Analyzer (Applied Biosystems) was utilized to read the sequences. The software programs Chromas Lite (v2.1.1) and BioEdit (v7.2.5) were used for sequence analysis.

### 4.4. Array-Comparative Genomic Hybridization (aCGH)

aCGH analyses were carried out using the oligonucleotide-based SurePrint G3 Human Genome CGH + SNP Microarray kit 4 × 180 K (Agilent Technologies, CA, USA), as described by the manufacturer instructions. Briefly, 1 µg of reference DNA (Agilent Euro Male/Female) and patient DNA were digested and labeled using the SureTag DNA Labeling kit (Agilent Technologies). Fragmented DNAs were labeled with Cy3 (reference DNA) and Cy5 (test samples) fluorescent dUTP, respectively. Purification columns were used to remove the unincorporated nucleotides and dyes. 

After purification, labeled sample and reference DNA were co-hybridized at 65 °C at 20 rpm for 24 h on the array slides, and scanned with Agilent G2565CA Microarray Scanner System. Features were normalized and extracted using feature extraction software (v11.1). Data were analyzed and visualized by Cytogenomics software (v2.7). For calling genomic imbalances, we applied the statistical algorithm ADM-2 with a sensitivity threshold of 6.0. A minimum of three-probe for aberration was used for filter. The log2 ratio of Cy3:Cy5 signal intensities was used by the program to consider a variation as a loss or a gain, with reasons ≤ −0.25 reasons considered loss. The log2 ratio of < −1.0 at the region of interest was considered to represent a homozygous deletion. 

### 4.5. Fluorescence In Situ Hybridization (FISH)

Cell suspensions were obtained from primary cultures using standard techniques (hypotonic treatment and methanol/acetic acid fixation). Interphase FISH (iFISH) was used to confirm and validate the aCGH results. In addition, this approach was used in samples which the aCGH analyses was not possible. Dual color iFISH experiments with commercially available probes were used according to standard procedures and/or to manufacturer’s instructions: TP53/CEN 17 Dual Color Probe (ZytoVision GmbH, Germany), PTEN/CEN 10 Dual Color Probe (ZytoVision GmbH, Germany), CDKN2A/CEN 9 Dual Color Probe (ZytoVision GmbH, Germany). Cells were counterstained with DAPI (4′,6-diamidino-2-phenylindole) and the slides were analyzed using a fluorescence microscope (AxioImager.Z1 mot, Zeiss) equipped with appropriate filter sets. The images were captured by a sensitive CCD camera and the result was processed by ISIS imaging system (MetaSystems, Altlußheim, Germany). In each sample, hybridization signals of at least 200 tumor cell interphase nuclei were counted. When ≥10% of the nuclei showed a decreased number of fluorescent signals of locus specific probe with respect to normal diploid cells, it was considered a loss event. For statistical value analyses, monosomy was included as heterozygous allelic loss.

### 4.6. Statistical Analysis

Microsoft Excel and Bioestat 5.3 were used to annotate information in the form of databases and to apply descriptive statistics, respectively. To compare the frequencies and mean scores of each group, noting possible statistical differences, parametric statistics tests (analysis of variance, student’s *t*-test) and non-parametric statistics tests (Fisher exact, chi-square, binomial proportion, poisson) were used, respectively. Additionally, simple logistic regression and partial correlation test were evaluated to reveal correlation between molecular alterations and variables of the experiments. The results were considered statistically significant when *p* values were ≤ 0.05.

## Figures and Tables

**Table 1:** Comparison of World Health Organization (WHO) grade of malignancy x age.

WHO Histological Grading	Mean Age	Significance (*p* Value)
**I**	11.8	Median I and II (*p* > 0.05) Median I and III (*p* < 0.05) Median I and IV (*p* < 0.05)
**II**	23.6	Median II and III (*p* < 0.05) Median II and IV (*p* < 0.05)
**III**	42.8	Median III and IV (*p* > 0.05)
**IV**	51.7	-

**Table 2:** *TP53* mutated gliomas.

Sample	Tumor	WHO Grading	Gender	Age	Exon
**CNS 1**	Subependymal giant cell astrocytoma	I	M	23	11
**CNS 14**	Glioblastoma	IV	F	68	5, 10
**CNS 31**	Analastic astrocytoma	III	F	65	4, 5
**CNS 98**	Diffuse astrocytoma	II	M	26	7
**CNS 101**	Glioblastoma	IV	M	79	5
**CNS 167**	Diffuse astrocytoma	II	M	14	7

**Table 3:** Frequency of *TP53* deletion in each histologic and WHO grading group.

*TP53*	All Cases		A-I		A-II		A-III		A-IV		OD-II		OD-III		OA-II		OA-III		ED-II		ED-III	
	N	%	N	%	N	%	N	%	N	%	N	%	N	%	N	%	N	%	N	%	N	%
Intact	46	70.8	5	55.6	9	90	4	66.7	16	66.7	3	100	0	0	2	100	1	50	3	75	3	75
Hom. Del	0	0	0	0	0	0	0	0	0	0	0	0	0	0	0	0	0	0	0	0	0	0
Het. Del	19	29.2	4	44.4	1	10	2	33.3	8	33.3	0	0	1	100	0	0	1	50	1	25	1	25
Total	65		9		10		6		24		3		1		2		2		4		4	

Hom. Del, homozygous deletion; Het. Del, heterozygous deletion; A, astrocytoma; OD, oligodendroglioma, OA, oligoastrocytoma, ED, ependymoma.

**Table 4:** Frequency of *PTEN* deletion in each histologic and WHO grading group.

*PTEN*	All Cases		A-I		A-II		A-III		A-IV		OD-II		OD-III		OA-II		OA-III		ED-II		ED-III	
	N	%	N	%	N	%	N	%	N	%	N	%	N	%	N	%	N	%	N	%	N	%
Intact	39	62.9	8	100	7	77.8	5	83.3	6	26.1	2	66.7	0	0	2	100	1	50	4	100	4	100
Hom. Del	0	0	0	0	0	0	0	0	0	0	0	0	0	0	0	0	0	0	0	0	0	0
Het. Del	23	37.1	0	0	2	22.2	1	16.7	17	73.9	1	33.3	1	100	0	0	1	50	0	0	0	0
Total	62		8		9		6		23		3		1		2		2		4		4	

Hom. Del, homozygous deletion; Het. Del, heterozygous deletion; A, astrocytoma; OD, oligodendroglioma, OA, oligoastrocytoma, ED, ependymoma.

**Table 5:** Frequency of *CDKN2A* deletion in each histologic and WHO grading group.

*CDKN2A*	All Cases		A-I		A-II		A-III		A-IV		OD-II		OD-III		OA-II		OA-III		ED-II		ED-III	
	N	%	N	%	N	%	N	%	N	%	N	%	N	%	N	%	N	%	N	%	N	%
Intact	35	55.6	6	75	4	44.4	3	50	10	41.7	2	66.7	0	0	2	100	1	50	4	100	3	75
Hom. Del	8	12.7	0	0	2	22.2	1	16.7	5	20.8	0	0	0	0	0	0	0	0	0	0	0	0
Het. Del	20	31.7	2	25	3	33.3	2	33.3	9	37.5	1	33.3	1	100	0	0	1	50	0	0	1	25
Total	63		8		9		6		24		3		1		2		2		4		4	

Hom. Del, homozygous deletion; Het. Del, heterozygous deletion; A, astrocytoma; OD, oligodendroglioma, OA, oligoastrocytoma, ED, ependymoma.

**Table 6:** Frequency of single and concurrent *TP53*, *PTEN,* and *CDKN2A* alteration by WHO grade of malignancy.

Gene Alterations	WHO Grading			
	Grade I	Grade II	Grade III	Grade IV
**One Gene**				
TP53 */PTEN/CDKN2A	1/8 (12.5%)	1/18 (5.6%)	2/13 (15.4%)	2/23 (8.7%)
TP53/PTEN */CDKN2A	-	1/18 (5.6%)	-	5/23 (21.7%)
TP53/PTEN/CDKN2A*	-	4/18 (22.2%)	1/13 (7.7%)	1/23 (4.3%)
**Two Genes**				
TP53*/PTEN */CDKN2A	-	-	-	1/23 (4.3%)
TP53*/PTEN/CDKN2A*	2/8 (25%)	-	2/13 (15.4%)	1/23 (4.3%)
TP53/PTEN */CDKN2A*	-	1/18 (5.6%)	2/13 (15.4%)	6/23 (26.1%)
**Three Genes**				
TP53*/PTEN */CDKN2A*	-	1/18 (5.6%)	1/13 (7.7%)	5/23 (21.7%)

* alterated; - no alteration.

**Table 7:** Clinical data, molecular, and cytogenetic findings of patient’s samples used in the study.

Patient	Histopathologic	WHO	Sex	Age	SSCP		aCGH + iFISH		
					*TP53*	*PTEN*	*TP53*	*PTEN*	*CDKN2A*
1	SGCA	1	M	23	mut	wt	x	x	x
2	DA	2	F	12	wt	wt	-	x	x
3	PA	1	M	13	wt	x	x	x	x
4	GBM	4	F	68	mut	x	no del	no del	no del
5	GBM	4	F	64	wt	x	-	-	- -
6	PMA	2	M	3	wt	wt	no del	no del	no del
7	GBM	4	M	65	wt	x	-	-	no del
8	OA	2	M	2	wt	wt	no del	no del	no del
9	AA	3	F	55	mut	wt	-	no del	-
10	GBM	4	F	7	wt	x	no del	x	- -
11	ED	2	F	2	x	x	-	no del	no del
12	AA	3	M	60	wt	wt	no del	no del	-
13	GSA	4	M	64	wt	wt	no del	-	no del
14	DA	2	M	37	wt	x	no del	no del	-
15	GBM	4	M	43	wt	wt	no del	no del	no del
16	ED	2	F	1	wt	wt	no del	no del	no del
17	DA	2	F	52	wt	x	no del	no del	no del
18	AOA	3	F	63	wt	wt	-	no del	no del
19	ED	2	M	8	wt	wt	no del	no del	no del
20	OD	2	M	19	wt	wt	no del	no del	no del
21	OD	2	F	43	wt	wt	no del	-	no del
22	DA	2	M	26	mut	x	x	x	x
23	GBM	4	M	79	mut	x	-	-	- -
24	AED	3	F	54	wt	wt	-	no del	no del
25	GBM	4	M	15	wt	x	-	-	-
26	SGCA	1	M	20	wt	x	-	no del	-
27	AA	3	F	23	x	x	no del	no del	no del
28	PA	1	F	16	wt	wt	-	no del	-
29	AOD	3	M	61	x	x	-	-	-
30	PXA	2	M	26	wt	wt	no del	no del	- -
31	PA	1	F	3	wt	x	-	x	x
32	PMA	2	F	2	x	x	no del	no del	no del
33	GBM	4	F	70	wt	wt	no del	-	-
34	DA	2	F	34	wt	wt	no del	no del	no del
35	GBM	4	M	51	wt	wt	no del	-	-
36	DA	2	M	64	wt	x	x	x	x
37	ED	2	F	8	wt	wt	no del	no del	no del
38	AED	3	M	62	wt	wt	no del	no del	-
39	PA	1	F	27	wt	wt	no del	no del	no del
40	AA	3	F	31	wt	wt	-	no del	- -
41	GBM	4	M	60	wt	wt	no del	-	- -
42	GBM	4	M	43	wt	wt	-	no del	- -
43	GBM	4	F	13	x	x	no del	-	no del
44	DA	2	M	14	mut	x	no del	-	-
45	GBM	4	M	46	x	x	no del	-	-
46	GBM	4	F	49	x	x	no del	-	no del
47	OD	2	F	23	x	x	no del	no del	-
48	AA	3	M	59	x	x	no del	-	-
49	GBM	4	M	56	x	x	-	-	-
50	GBM	4	M	74	wt	wt	no del	no del	-
51	GBM	4	M	48	wt	wt	no del	-	no del
52	GBM	4	M	32	x	x	-	-	-
53	PA	1	M	7	wt	wt	no del	no del	no del
54	AED	3	M	19	wt	wt	no del	no del	no del
55	PA	1	F	9	x	x	no del	no del	no del
56	GSA	4	M	77	wt	wt	-	no del	no del
57	PA	1	M	2	wt	wt	no del	no del	no del
58	DA	2	M	65	wt	wt	no del	-	-
59	AOA	3	M	24	wt	wt	no del	-	-
60	AA	3	M	58	x	x	no del	no del	no del
61	OA	2	M	3	wt	wt	no del	no del	no del
62	DA	2	M	31	x	x	no del	no del	- -
63	GBM	4	M	33	x	x	no del	no del	no del
64	PA	1	F	4	x	x	no del	no del	no del
65	AED	3	M	2	x	x	no del	no del	no del
66	PA	1	F	6	x	x	-	no del	no del
67	GBM	4	F	66	x	x	no del	-	-
68	GBM	4	M	43	x	x	no del	-	no del
69	GBM	4	F	74	x	x	no del	-	no del

AA, anaplastic astrocytoma; AED, anaplastic ependymoma; AOA, anaplastic oligoastrocytoma; AOD, anaplastic oligodendroglioma; DA, diffuse astrocytoma; ED, ependymoma; FA, fibrillary astrocytoma; GBM, glioblastoma; GSA, gliosarcoma; OA, oligoastrocytoma; OD, oligodendroglioma; PA, pilocytic astrocytoma; PMA, pilomyxoid astrocytoma; PXA, pleomorphic xanthoastrocytoma; SGCA, subependymal giant cell astrocytomas; mut, mutated, wt, wild type; x, not available; no del, no deletion.

**Table 8:** Primers used for *TP53* gene.

Exon	Primer	Primer Lenght (bp)	Exon Lenght (pb)	Annealing Temperature (°C)
Exon 4	**F**-5′ TTTCACCCATCTACAGTCCC 3′ **R**-5′ CATTGAAGTCTCATGGAAGC 3′	20 20	279	54
Exon 5	**F**-5′ TTCACTTGTGCCCTGACTT 3′ **R**-5′ AACCAGCCCTGTCCGTCTC 3′	19 19	184	57
Exon 6	**F**-5′ CAGGGCTGGTTGCCCAGGGTCCCCC 3′ **R**-5′ ACTGACAACCACCCTTAACCCCTCC 3′	25 25	113	59
Exon 7	**F**-5′ TGCTTGCCACAGGTCT 3′ **R**-5′ ACAGCAGGCAGTGT 3′	16 14	110	54
Exon 8	**F**-5′ CCACCGCTTCTTGTCCTGC 3′ **R**-5′ CCTACTGCCTCTTTGCTTC 3′	19 19	137	58
Exon 9	**F**-5′ AGGGTGCAGTTATGCCTCAG 3′ **R**-5′ ACTTGATAAGAGGTCC 3′	20 16	74	52
Exon 10	**F**-5′ CTGAGGCACAAGAATCAC 3′ **R**-5′ TCCTATGGCTTTCCAACC 3′	18 18	107	59
Exon 11	**F**-5′ GGAAGGGTCAACATCTTTTACA 3′ **R**–5′ TAAAAAGGGAGAAGGAGGGG 3′	22 20	1289	59

**Table 9:** Primers used for *PTEN* gene.

Exon	Primer	Primer Lenght (bp)	Exon Lenght (pb)	Annealing Temperature (°C)
Exon 5	**F**-5′ ACCTGTTAAGTTTGTATGCAAC 3′ **R**-5′ TCTGTTTTCCAATAAATTCTC 3′	22 21	239	51
Exon 6	**F**-5′ CTACGACCCAGTTACCATAGCA 3′ **R**-5′ GGCTTCTTTAGCCCAATGAGTTG 3′	22 23	142	59
Exon 7	**F**-5′ TGACAGTTTGACAGTTAAAGG 3′ **R**-5′ CTCCCAATGAAAGTAAAGTACA 3′	21 22	167	53
